# Design of a 3D BMP-2-Delivering Tannylated PCL Scaffold and Its Anti-Oxidant, Anti-Inflammatory, and Osteogenic Effects In Vitro

**DOI:** 10.3390/ijms19113602

**Published:** 2018-11-15

**Authors:** Jae Yong Lee, Hyunwoong Lim, Jae Won Ahn, Dongsik Jang, Seung Hee Lee, Kyeongsoon Park, Sung Eun Kim

**Affiliations:** 1 Department of Orthopedic Surgery and Rare Diseases Institute, Korea University Guro Hospital, Korea University College of Medicine, 148 Guro-dong, Guro-gu, Seoul 08308, Korea; bulegir@nate.com; 2 Department of Industrial Management Engineering, Korea University, 145 Anam-ro, Seongbuk-gu, Seoul 02841, Korea; limhw@korea.ac.kr (H.L.); Jang@korea.ac.kr (D.J.); 3 Department of Systems Biotechnology, Chung-Ang University, Anseong, Gyeonggi-do 17546, Korea; pinkymonkey1@naver.com; 4 Department of Nursing, Inha University, 100 Inha-ro, Michuhol-gu, Incheon 22212, Korea; freelovein@hanmail.net

**Keywords:** bone morphogenic protein-2 (BMP-2), tannic acid (TA), polycaprolactone (PCL), 3D scaffold, anti-oxidation, anti-inflammation, osteogenic differentiation

## Abstract

In this study, a novel three-dimensional (3D) bone morphogenic protein-2 (BMP-2)-delivering tannylated polycaprolactone (PCL) (BMP-2/tannic acid (TA)/PCL) scaffold with anti-oxidant, anti-inflammatory, and osteogenic activities was fabricated via simple surface coating with TA, followed by the immobilization of BMP-2 on the TA-coated PCL scaffold. The BMP-2/TA/PCL scaffold showed controlled and sustained BMP-2 release. It effectively scavenged reactive oxygen species (ROS) in cells, and increased the proliferation of MC3T3-E1 cells pre-treated with hydrogen peroxide (H_2_O_2_). Additionally, the BMP-2/TA/PCL scaffold significantly suppressed the mRNA levels of pro-inflammatory cytokines, including matrix metalloproteinases-3 (MMP-3), cyclooxygenase-2 (COX-2), interleukin-6 (IL-6), and tumor necrosis factor-α (TNF-α), in lipopolysaccharide (LPS)-induced MC3T3-E1 cells. Furthermore, it showed outstanding enhancement of the osteogenic activity of MC3T3-E1 cells through increased alkaline phosphatase (ALP) activity and calcium deposition. Our findings demonstrated that the BMP-2/TA/PCL scaffold plays an important role in scavenging ROS, suppressing inflammatory response, and enhancing the osteogenic differentiation of cells.

## 1. Introduction

Three-dimensional (3D) scaffolds fabricated by 3D printing techniques have been developed as bone grafts to effectively promote the repair of large bone defects because they are essential for cell proliferation and differentiation for tissue regeneration. Various 3D printing techniques, including 3D printing, solid freeform fabrication, and rapid prototyping, have emerged to prepare versatile and detailed biomimetic 3D scaffolds with high precision and accuracy by controlling pore size, interconnectivity, porosity, and mechanical strength [1,2,3,4,5]. Although 3D scaffolds are excellent templates for bone tissue regeneration, scaffolds alone cannot satisfactorily improve the proliferation and differentiation of cells during bone formation.

Bone plays an important role in the support and protection of the various organs of the body. Additionally, it is a dynamic tissue that experiences renewal and repair all through life via the process of bone remodeling. During the regulation of bone remodeling, osteoblast and osteoclast activities continually support osteogenesis and bone resorption sequences. However, several previous studies reported that oxidative stresses, such as reactive oxygen species (ROS), restrained osteoblast differentiation and induced apoptosis [6,7,8]. The over-expression of ROS in osteoblasts was associated with the pathophysiology of bone loss [9,10,11], suppressing mineralization and delaying bone repair.

Scaffold functionalization techniques with osteoinductive molecules have been developed to accelerate and enhance osteogenic differentiation and bone tissue regeneration [12,13,14,15]. Dopamine has been widely used for surface functionalization of scaffolds because it can be easily immobilized on the versatile inorganic and organic substrates [16]. However, dopamine self-polymerization has several limitations, such as interference of protein/peptide quantification, the reduction of drug activity, and polymer stability under alkaline conditions [17]. Additionally, the effect of ROS scavenging on polydopamine coating is unclear in cells. We have recently developed heparin-immobilized scaffolds using heparin-dopamine (Hep-DOPA) to effectively deliver osteoinductive growth factors for enhancing bone tissue regeneration [15,18,19]. Although Hep-DOPA coating on scaffolds is effective in controlling growth factor release from the scaffolds for a long period, the synthesis and purification of Hep-DOPA should be preceded before it is coated on the surface of the scaffolds.

Plant phenols and polyphenols, such as tannic acid (TA), quercetin, 5-pyrogallol 2-aminoethane, and epigallocatechin gallate, have been used for the surface functionalization of substrates because they can easily be deposited on various organic and inorganic substrates via covalent and non-covalent bonds [20]. Among various polyphenols, TA, with a central glucose molecule containing multiple hydroxyl groups, is used as a promising platform for surface functionalization using various biomacromolecules (i.e., DNA, gelatin, collagen, albumin, chitosan, thrombin, and mucin) due to its capability of multiple interactions with various molecules via charge-to-charge, hydrogen bond, and/or hydrophobic interactions [17,21,22]. More importantly, TA has pleiotropic effects, such as anti-oxidant, anti-inflammatory, anti-bacterial, and anti-cancer properties [23,24]. In particular, TA is effective in inhibiting lipid oxidation and radical-mediated DNA cleavage by scavenging oxygen and oxygen-derived radicals [24,25,26], and TA-containing agarose hydrogels have exhibited an enhanced anti-inflammatory effect [27].

Bone morphogenic proteins (BMPs) have been widely used because they play important roles in bone tissue regeneration. Among various BMPs, BMP-2, as a potent osteoinductive growth factor, significantly stimulates osteogenic differentiation of mesenchymal stem cells and enhances bone formation [28,29,30]. Despite the effectiveness on osteogenic differentiation and bone formation, the delivery of BMP-2 alone is not effective in the bone formation of defect sites due to its short biological half-life through rapid diffusion by body fluid and clearance [31]. Therefore, BMP-2 should be incorporated or immobilized within/on the substrates to maintain the controlled release and improve its therapeutic efficacy.

Based on these previous reports, in this study, we designed a new 3D polycaprolactone (PCL) scaffold with anti-oxidant, anti-inflammatory, and osteogenic properties by mixing a scaffold with TA and BMP-2. To facilitate anti-oxidant and anti-inflammatory effects, the surface of the PCL scaffold was coated with TA. Additionally, to enhance osteogenic activity, BMP-2 was immobilized on TA-coated PCL scaffolds. Then, we evaluated in vitro anti-oxidant and anti-inflammatory effects and osteogenic activity of the scaffolds with TA and BMP-2.

## 2. Results

### 2.1. Preparation and Characterizations of PCL and the Functionalized PCL Scaffolds with TA and/or BMP-2

**Figure 1:**
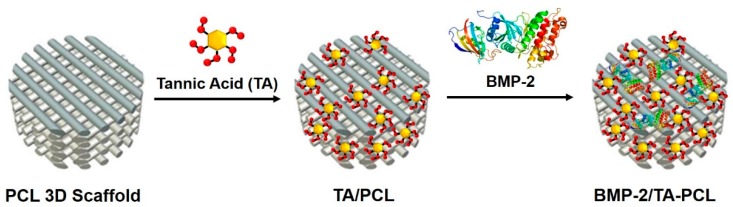
Schematic illustration for the preparation of the bone morphogenic protein-2 (BMP-2)/tannic acid (TA)/three-dimensional (3D) polycaprolactone (PCL) scaffold.

In this study, we prepared 3D PCL scaffolds via a layer-by-layer process using a 3D printing technique. The surface of the fabricated 3D PCL scaffolds was then coated with TA, followed by BMP-2 immobilization on PCL or TA-coated PCL (TA/PCL) scaffolds to yield BMP-2/PCL or BMP-2/TA/PCL scaffolds (Figure 1).

**Figure 2:**
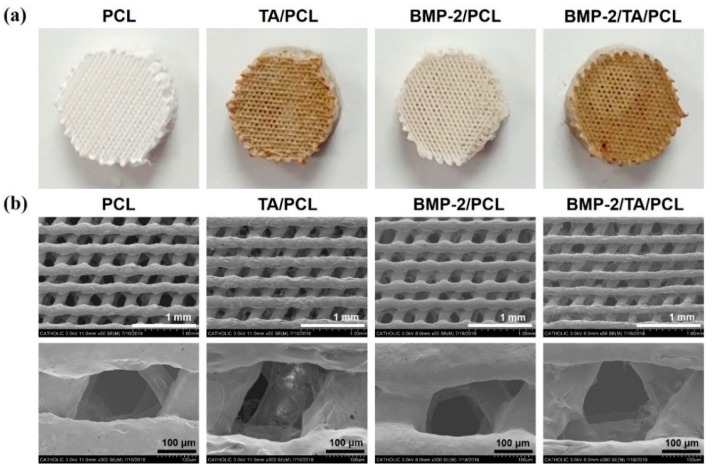
(**a**) Photos and (**b**) scanning electron microscopy (SEM) images of PCL, TA/PCL, BMP-2/PCL, and BMP-2/TA/PCL.

The coated amount and efficiency of TA on TA/PCL was 6.19 ± 0.86 µg and 61.93 ± 8.56%, respectively. Additionally, the immobilized amount of BMP-2 was 262.99 ± 13.34 ng for BMP-2/PCL and 388.38 ± 10.36 ng for BMP-2/TA/PCL. The immobilization efficiency of BMP-2 was 52.59 ± 2.67% for BMP-2/PCL and 77.67 ± 2.07% for BMP-2/TA/PCL. The TA coating on the PCL scaffolds was confirmed with Ag deposition. TA/PCL and BMP-2/TA/PCL showed light brown colors due to Ag deposition on the PCL scaffolds, whereas bare PCL and BMP-2/PCL showed no color change (Figure 2a). To further verify the TA coating and BMP-2 immobilization on the PCL scaffolds, the elemental chemical compositions of each scaffold were determined by X-ray photoelectron spectroscopy (XPS) analysis (Table 1). The TA/PCL scaffold displayed an increased O1s content (1.23%) compared with the PCL. After BMP-2 immobilization on PCL or TA/PCL scaffolds, the nitrogen content (N1s) increased compared with the PCL (from 0 to 0.64% or from 0 to 1.28%, respectively), indicating that BMP-2/TA/PCL has relatively more BMP-2 protein on the scaffold surface compared to BMP-2/PCL. All scaffolds, including PCL, TA/PCL, BMP-2/PCL, and BMP-2/TA/PCL, possessed well-defined porous structures.

**Figure 3:**
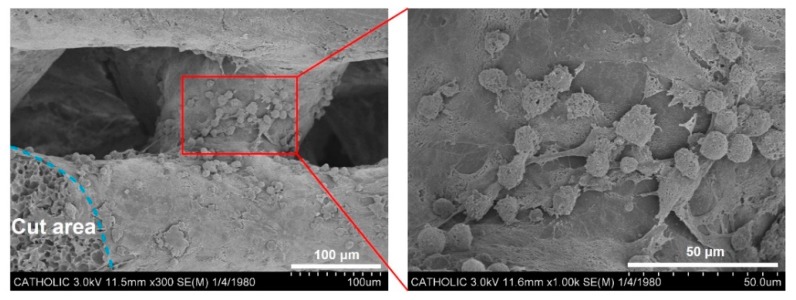
SEM images of the infiltrated cells inside 3D PCL scaffolds.

Scanning electron microscopy (SEM) images showed that all scaffolds have similar and regular pore sizes, structures, and thicknesses of each layer, relatively smooth surfaces, and the pores in all groups were connected to each other (Figure 2b). The determined thicknesses of each layer for PCL, TA/PCL, BMP-2/PCL, and BMP-2/TA/PCL were 190.67 ± 5.28 µm, 167.60 ± 0.21 µm, 170.43 ± 7.30 µm, and 148.46 ± 7.21 µm, respectively. The thickness in each layer of the scaffolds might have varied slightly during the drying step following solvent removal. Additionally, the coating of the scaffolds with TA and/or BMP-2 may not have changed the thickness of each layer because the used amounts of TA and BMP-2 were very small. The observed pore sizes of the scaffolds in all groups were over 150 μm and the pores were interconnected. These interconnected pores in scaffolds facilitate the transport of biomolecules and cells. After seeding mouse osteoblastic MC3T3-E1 cells on the scaffolds, the seeded cells were infiltrated, attached and proliferated inside the scaffolds (Figure 3).

### 2.2. In Vitro Release of BMP-2 from Scaffolds

**Figure 4:**
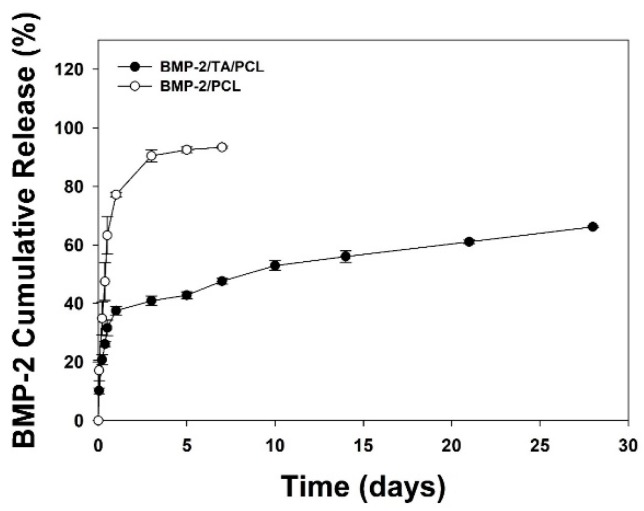
In vitro cumulative BMP-2 release profiles for the BMP-2/PCL and BMP-2/TA/PCL scaffolds.

Figure 4 shows the in vitro BMP-2 release profiles from BMP-2/PCL and BMP-2/TA/PCL. BMP-2/PCL showed a much faster BMP-2 release than BMP-2/TA/PCL. The BMP-2/PCL group released BMP-2 up to 77.18 ± 0.76% on day 1 and 93.38 ± 0.38% on day 7. However, BMP-2/TA/PCL released BMP-2 up to 37.51 ± 1.47% on day 1, 47.62 ± 0.99% on day 7, and 66.19 ± 0.30% on day 28.

### 2.3. In Vitro Anti-Oxidant Study

**Figure 5:**
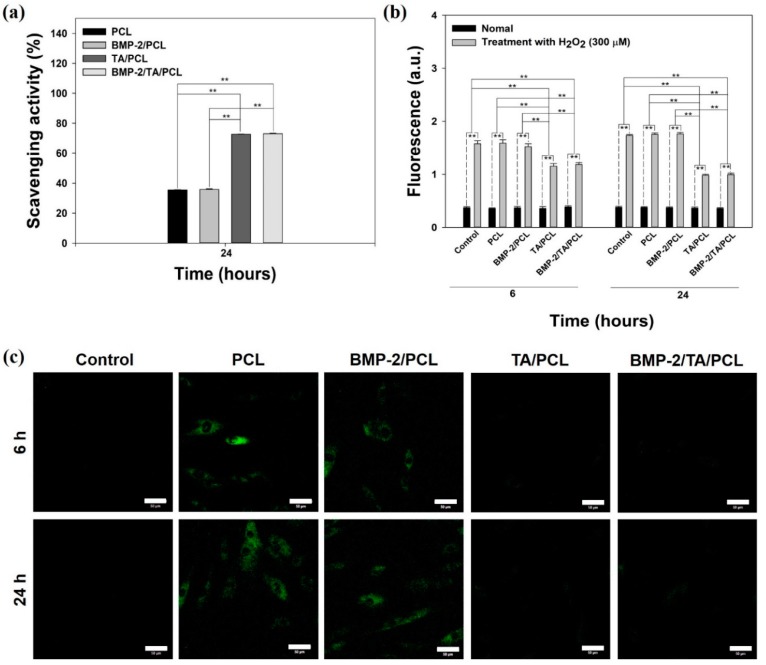
(**a**) Anti-oxidant activities of PCL, TA/PCL, BMP-2/PCL, and BMP-2/TA/PCL scaffolds measured by a 2,2-diphenyl-1-picryhydrazyl (DPPH) assay. (**b**) Quantitative intracellular ROS levels of MC3T3-E1 cells treated with the extract from the PCL, TA/PCL, BMP-2/PCL, and BMP-2/TA/PCL scaffolds for 6 h or 24 h after the cells were treated with 300 µM H_2_O_2_ for 30 min. Error bars represent mean ± SD, ** *p* < 0.01. (**c**) Fluorescence images of intracellular levels of MC3T3-E1 cells treated with the extract from the PCL, TA/PCL, BMP-2/PCL, and BMP-2/TA/PCL scaffolds for 6 h or 24 h after the cells were treated with 300 µM H_2_O_2_ for 30 min. After 6 h or 24 h treatment, the cells were stained with 2’,7-dichlorodihydrofluorescein diacetate (DCFDA) and observed by a confocal laser scanning microscope (CLSM). Scale bar = 50 μm.

The in vitro anti-oxidant activities of the extracts from each scaffold were screened by 2,2-diphenyl-1-picryhydrazyl (DPPH, λ_max_ = 517 nm) assay. As shown in Figure 5a, the PCL and BMP-2/PCL scaffolds were not effective in DPPH scavenging. However, the TA/PCL and BMP-2/TA/PCL groups exhibited much higher DPPH scavenging up to approximately 73% and stronger anti-oxidant activities than the PCL and BMP-2/PCL groups, indicating that TA-coated scaffolds, including TA/PCL and BMP-2/TA/PCL, have anti-oxidant potential.

### 2.4. ROS Scavenging Effects in Cells

To further demonstrate in vitro anti-oxidant activities of the scaffolds, the MC3T3-E1 cells pre-treated with 300 µM hydrogen peroxide (H_2_O_2_) were treated with the extracts from each scaffold for 6 h and 24 h, and then, ROS levels of each group were measured with 2’,7-dichlorodihydrofluorescein diacetate (DCFDA) fluorescence intensity and images in cells. Under normal conditions, all groups showed similar fluorescence intensities (Figure 5b). However, under the 300-µM H_2_O_2_ condition, PCL and BMP-2/PCL exhibited higher and similar fluorescence intensities compared to the control group, indicative of no anti-oxidant activities. However, treatments of the extracts from TA/PCL and BMP-2/TA/PCL for 6 h and 24 h could significantly decrease fluorescence intensities, indicative of excellent anti-oxidant activities. Consistent with these results, under normal conditions, the untreated cells (without both exogenous H_2_O_2_ and the extracts from the scaffolds) showed no fluorescence intensities, whereas the cells treated with the extracts from PCL and BMP-2/PCL showed strong fluorescence signals under the ROS condition (Figure 5c). However, the fluorescence signals in cells treated with the extracts from TA/PCL and BMP-2/TA/PCL were not observed, indicating that TA/PCL and BMP-2/TA/PCL are very effective in decreasing ROS levels in cells.

### 2.5. Protection of Cell Viabilities against the ROS Condition

**Figure 6:**
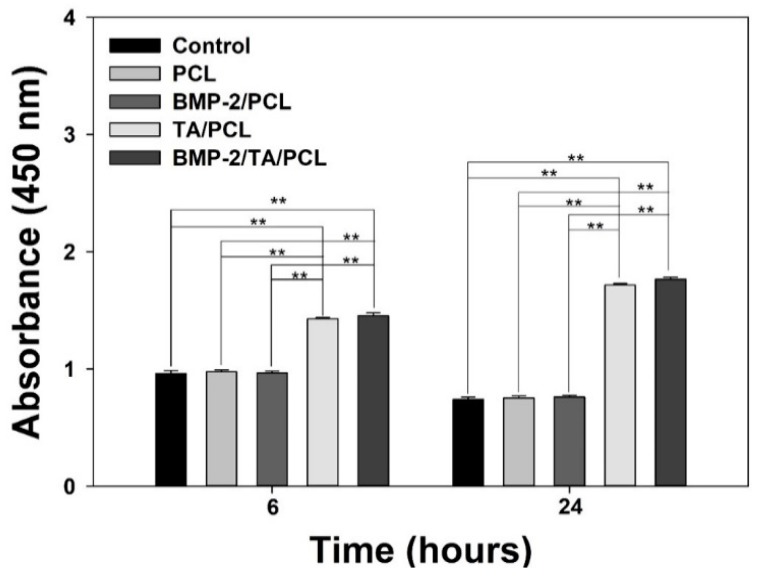
Cell viabilities of MC3T3-E1 cells grown on PCL, TA/PCL, BMP-2/PCL, and BMP-2/TA/PCL at 6 h and 24 h after the cells were pre-treated with 300 µM H_2_O_2_. Error bars represent mean ± SD, ** *p* < 0.01.

In order to investigate whether the scaffolds are effective in the protection of cells against the 300 µM H_2_O_2_ condition, the proliferation of MC3T3-E1 cells grown on each scaffold were measured at 6 h and 24 h after the cells were pre-treated with 300 µM H_2_O_2_. As shown in Figure 6, under the 300-µM H_2_O_2_-treated condition, the PCL and BMP-2/PCL groups showed similar cell viabilities at 6 h compared to the control group, and the cell viabilities in these groups were decreased at 24 h. However, at 6 h, cell viabilities of the cells grown on the TA/PCL and BMP-2/TA/PCL groups were much higher than those on the control, PCL, and BMP-2/PCL (** *p* < 0.01). Interestingly, the cells grown on the TA/PCL and BMP-2/TA/PCL groups were more significantly proliferated at 24 h than at 6 h. This indicates that anti-oxidant activities of the TA/PCL and BMP-2/TA/PCL groups effectively protect the cells from the toxic ROS environment, leading to increasing cell proliferation.

### 2.6. Anti-Inflammatory Effects of the Scaffolds on Lipopolysaccharide-Stimulated MC3T3-E1 Cells

**Figure 7:**
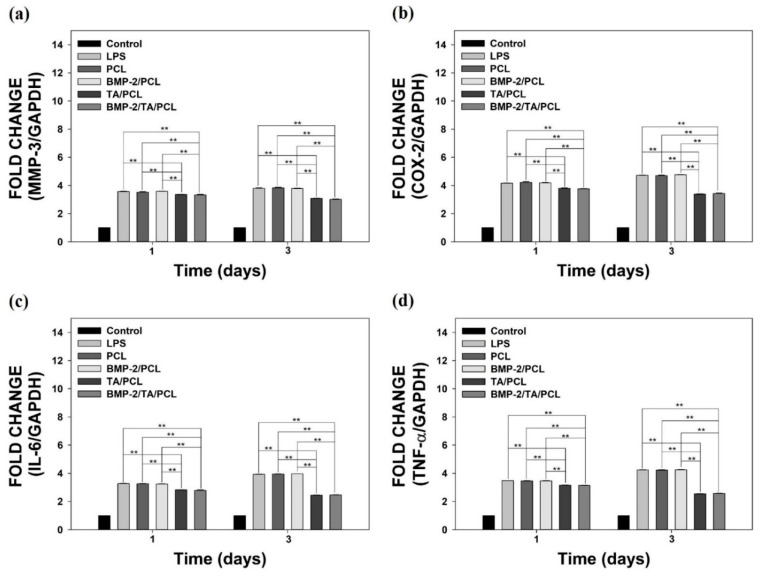
The relative mRNA expression levels of pro-inflammatory cytokines, including (**a**) matrix metalloproteinases-3 (MMP-3), (**b**) cyclooxygenase-2 (COX-2), (**c**) interleukin-6 (IL-6), and (**d**) tumor necrosis factor-α (TNF-α) in lipopolysaccharide (LPS)-stimulated MC3T3-E1 cells grown on PCL, TA/PCL, BMP-2/PCL, and BMP-2/TA/PCL scaffolds on day 1 and day 3. Error bars represent the mean ± SD. ** *p* < 0.01.

To evaluate the in vitro anti-inflammatory effects of the scaffolds on lipopolysaccharide (LPS)-stimulated MC3T3-E1 cells, the mRNA levels of pro-inflammatory cytokines, including matrix metalloproteinases-3 (MMP-3), cyclooxygenase-2 (COX-2), interleukin-6 (IL-6), and tumor necrosis factor-α (TNF-α), were determined by real-time polymerase chain reaction (PCR) on day 1 and day 3 (Figure 7). The LPS-treated cells showed the highest mRNA levels of pro-inflammatory cytokines on day 1 and day 3. The PCL and BMP-2/PCL groups did not suppress the mRNA levels of these pro-inflammatory cytokines compared to those in the LPS-treated group, suggesting that PCL and BMP-2/PCL have no anti-inflammatory effects. However, TA/PCL and BMP-2/TA/PCL significantly decreased the mRNA levels of MMP-3, COX-2, IL-6, and TNF-αin LPS-treated cells compared to those in the other groups (** *p* < 0.01). These data imply that TA/PCL and BMP-2/TA/PCL can decrease inflammatory responses in LPS-treated cells.

### 2.7. Alkaline Phosphatase Activity

**Figure 8:**
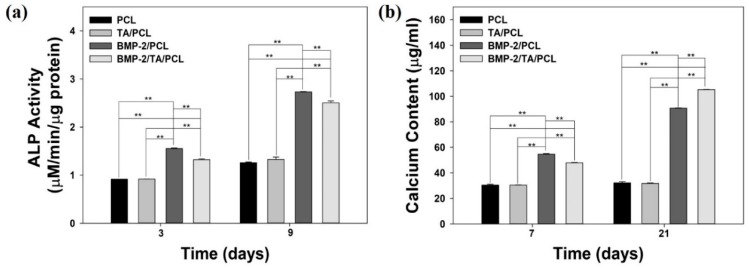
(**a**) Alkaline phosphatase (ALP) activity and (**b**) calcium deposition of MC3T3-E1 cells cultured on PCL, TA/PCL, BMP-2/PCL, and BMP-2/TA/PCL scaffolds. Error bars represent the mean ± SD. ** *p* < 0.01.

The alkaline phosphatase (ALP) activities of MC3T3-E1 cells grown on each scaffold were measured on day 3 and day 9 (Figure 8a). At 3 and 9 days, the ALP activities of the MC3T3-E1 cells in the BMP-2/PCL and BMP-2/TA/PCL groups were significantly increased and much greater than those of the cells in PCL or TA/PCL (** *p* < 0.01). Interestingly, BMP-2/PCL showed slightly higher ALP activity than BMP-2/TA/PCL due to a faster release of BMP-2 from BMP-2/PCL compared to BMP-2/TA/PCL within 10 days. Meanwhile, no significant differences of the ALP activities between PCL and TA/PCL were observed at three days and nine days.

### 2.8. Calcium Deposition

The amount of calcium deposited by MC3TC-E1 cells grown on PCL, TA/PCL, BMP-2/PCL, and BMP-2/TA/PCL was analyzed after seven days and 21 days of culture (Figure 8b). On day seven and day 21, there were no significant differences of calcium content between PCL and TA/PCL. However, the deposited calcium contents of BMP-2/PCL and BMP-2/TA-PCL were much higher than those of PCL and TA/PCL (** *p* < 0.01) on day seven and day 21. On day seven, BMP-2/PCL showed slightly higher calcium content than BMP-2/TA/PCL due to faster BMP-2 release from BMP-2/PCL compared to BMP-2/TA/PCL within 10 days. In contrast, on day 21, BMP-2/TA/PCL exhibited higher calcium contents than BMP-2/PCL, indicating that the sustained BMP-2 release from BMP-2/TA/PCL for a long time continuously stimulate osteoblastic differentiation compared to BMP-2/PCL without showing sustained release of BMP-2. These data indicate that BMP-2-releasing scaffolds influenced significantly higher osteogenic activities than the scaffolds without BMP-2 (** *p* < 0.01).

## 3. Discussion

In this study, we designed a new 3D PCL scaffold with anti-oxidant, anti-inflammatory, and osteogenic activities for enhancing bone tissue regeneration. The 3D PCL scaffolds with anti-oxidant, anti-inflammatory, and osteogenic activities were fabricated by simple surface coating of the scaffold with TA, followed by the immobilization of BMP-2 as an osteoinductive growth factor on the TA-modified PCL scaffold to obtain the BMP-2/TA/PCL scaffold. We investigated the potential of the BMP-2/TA/PCL scaffold that can scavenge oxidative stress, suppress inflammation responses, and enhance osteogenic activities.

Recently, plant phenols and polyphenols (i.e., TA, quercetin, 5-pyrogallol 2-aminoethane, and epigallocatechin gallate) have been attractive to use as surface modifiers because they interact with each other via covalent and non-covalent bonds and deposit on organic and inorganic substrates [20]. Among them, TA coating is a particularly promising method as a versatile platform for surface functionalization because the TA molecule can interact with cationic molecules via electrostatic interactions, facilitate hydrogen bond interaction with other molecules, and mediate hydrophobic interactions by its multiple aromatic rings [17]. In this study, a 3D PCL scaffold was coated with TA, followed by the immobilization of BMP-2. PCL and modified PCL scaffolds with TA and/or BMP-2 showed uniformed pore size, structures, and thickness as previously reported [3,15]. TA coating on PCL scaffold was confirmed through the increased oxygen content due to a large number of galloyl residues with multiple phenolic hydroxyl groups in the TA molecule. Geissler et al. similarly reported that a TA precursor or TA-coated titanium disc had increased oxygen content [32]. Furthermore, it was reported that the TA molecule has a strong affinity with biomacromolecules, such as DNA, gelatin, collagen, albumin, chitosan, thrombin, and mucin, via multiple interactions, including electrostatic, hydrogen, and hydrophobic interactions [21,22]. Due to these multiple interactions between TA and biomacromolecules, the BMP-2/TA/PCL scaffold exhibited sustained and controlled release of BMP-2 from the scaffolds compared with the BMP-2/PCL scaffold without TA coating.

As a plant-derived polyphenol, TA has pleiotropic effects, such as anti-oxidant, anti-inflammatory, anti-mutagenic, anti-carcinogenic, anti-microbial, and anti-allergic properties [23,24]. TA consists of a central glucose and 10 galloyl residues, and its polyphenolic nature is the feature responsible for its anti-oxidant action [33]. Radical scavenging activities are very important because free radicals in foods and in biological systems are deleterious. To investigate the free radical scavenging activity of the scaffolds, a DPPH assay was used because the anti-oxidants could reduce the stable radical DPPH to the yellow-colored diphenyl-picryhydrazine [34]. Consistent with the previous study that found that gelatin nanofibers cross-linked with TA showed more anti-oxidant activity than gelatin nanofibers only [35], in the DPPH assay, TA/PCL and BMP-2/TA/PCL scaffolds showed more effective radical scavenging activities than PCL and BMP-2/PCL scaffolds, indicating that TA molecules on the scaffolds had an effective radical scavenging activity because the hydroxyl groups of the galloyl residues in TA react with DPPH to easily remove the free radicals.

Oxidative stress caused by an imbalance between the free radicals and anti-oxidant activity is associated with various diseases, including cardiovascular disease, osteoporosis, atherosclerosis, diabetes, and carcinogenesis [36,37,38]. As one ROS, hydrogen peroxide (H_2_O_2_), with strong oxidizing properties, is usually seen as an oxidative stress. H_2_O_2_ formed by many oxidizing enzymes (i.e., superoxide dismutase) can cross membranes, followed by slowly oxidizing a number of compounds. Additionally, the treatment of exogenous H_2_O_2_ into cells leads to intracellular ROS production. To determine the ability of the scaffolds to scavenge intracellular ROS, MC3T3-E1 cells were treated with exogenous 300 µM H_2_O_2_ to induce oxidative stress [39,40] and further treated with the extracts from the scaffolds. As seen in the DCFDA fluorescence assay and CLSM images, TA/PCL and BMP-2/TA/PCL could markedly decrease the fluorescence signals of DCFDA in cells compared to PCL and BMP-2/PCL. In contrast, H_2_O_2_ itself is not very reactive. However, it can sometimes be toxic to cells because it may induce hydroxyl radicals in the cells. In this study, we confirmed that the cell viabilities of H_2_O_2_-treated MC3T3-E1 cells were decreased in a time-dependent manner, due to oxidative damage to cellular components [41]. Additionally, PCL and BMP-2/PCL groups exhibited similar cell viabilities compared to H_2_O_2_-treated MC3T3-E1 cells. Interestingly, the TA/PCL and BMP-2/TA/PCL groups displayed much higher cell viabilities than the PCL and BMP-2/PCL groups, and increased cell proliferation in a time-dependent manner. This result suggests that TA molecules on the scaffolds effectively scavenge intracellular ROS and may protect cells from oxidative damage, leading to increasing cell viabilities and proliferation [41,42].

It is known that oxidative stress can cause inflammatory responses via the activation of redox sensitive transcription factors, such as NF-κB, that play a critical role in inflammatory response induction [43]. During the inflammatory responses, inflammatory factors are important in bone repair. The previous study reported that pro-inflammatory cytokines inhibited osteogenic differentiation from mesenchymal stem cells (MSC) [44]. Among them, TNF-α and IL-1β suppressed the osteogenic differentiation from the MSC by decreasing mRNA levels of several osteoblast markers such as ALP, a1(I) procollagen, runt-related transcription factor 2 (Runx2), and osterix. Thus, we further examined in vitro anti-inflammatory effects of the scaffolds on inflamed cells. To investigate the in vitro anti-inflammatory effects of the scaffolds, MC3T3-E1 cells were treated with LPS to mimic the in vitro inflammatory environment. The treatment of LPS to the cells upregulates certain cytokines, such as TNF-α, IL-6, and IL-1β, and these cytokines further stimulate the induction of pro- and anti-inflammatory cytokines (i.e., TNF-α, IL-6, IL-10, IL-1β, COX-2, and proteolytic enzymes) [45,46]. In this study, LPS-treated MC3T3-E1 cells markedly increased the mRNA levels of pro-inflammatory cytokines (MMP-3, COX-2, IL-6, and TNF-α) compared to normal cells. The mRNA levels of these cytokines were not decreased by the treatment of PCL and BMP-2/PCL. However, TA/PCL and BMP-2/TA/PCL could significantly suppress the expression of these cytokines in a time-dependent manner. These data suggest that the scaffolds (TA/PCL and BMP-2/TA/PCL) coated with TA molecules had an effective anti-inflammatory function, following the previously reported finding that a carboxylated agarose hydrogel with TA molecules had an enhanced anti-inflammatory effect [27]. We also think that the anti-inflammatory function of the TA/PCL and BMP-2/TA/PCL scaffolds may be associated with the effective radical scavenging activity.

Osteogenic properties of the scaffolds were evaluated by determining the expression of distinctive osteogenic markers, such as ALP (an early osteogenic marker) and calcium deposition (a late osteogenic marker) [14,47]. The ALP activities and calcium content in BMP-2/PCL and BMP-2/TA/PCL were significant higher than those in PCL and TA/PCL because the released BMP-2 from the scaffolds may influence the stimulation of these osteogenic genes during cell differentiation [14]. These results indicate that the scaffolds with BMP-2, a potent osteoinductive molecule, were more effective in enhancing osteogenic activities by inducing osteogenic differentiation of MC3T3-El cells than the scaffolds without BMP-2.

In the present study, we investigated the in vitro anti-oxidant, anti-inflammatory, and osteogenic properties of the scaffolds. TA coating on the scaffolds enhanced the anti-oxidant and anti-inflammatory functions of the scaffolds; BMP-2 immobilization on the scaffolds increased the osteogenic differentiation of the cells. Taken together, the fabricated BMP-2/TA/PCL scaffolds effectively protected cells from toxic ROS environments and inflammatory responses by scavenging radicals as well as enhancing the osteogenic differentiation of the cells. Therefore, BMP-2/TA/PCL fabricated by a simple surface functionalization method will have potential in bone tissue regeneration.

## 4. Materials and Methods

### 4.1. Materials

Polycaprolactone (PCL, Molecular weight (Mw): 70,000-90,000)), tannic acid (TA, Mw: 1701.20), dichloromethane (DCM), ascorbic acid, dexamethasone, and β-glycerophosphate were purchased from Sigma-Aldrich (St. Louis, MO, USA). *Escherichia coli*-derived recombinant bone morphogenic protein-2 (BMP-2) and BMP-2 enzyme-linked immunsorbent assay (ELISA kits) were obtained from Pepro Tech, Inc. (Rocky Hill, NJ, USA). Dulbecco’s modified Eagle’s medium (DMEM), fetal bovine serum (FBS), phosphate-buffered saline (PBS), and antibiotics were purchased from Gibco BRL (Rockville, MD, USA). The cell counting kit (CCK)-8 was purchased from Dojindo Molecular Technologies, Inc. (Tokyo, Japan). All other chemicals were of the purest analytical grade available.

### 4.2. Fabrication of PCL Scaffolds

3D PCL scaffolds were fabricated using a rapid prototyping (RP) method, following a procedure as described previously [3,15]. PCL (4 g) pellets were dissolved in 20 mL of acetone/dimethyl sulfoxide (DMSO) (1:1, *v*/*v*) co-solvents at 60 °C for 5 h. The PCL solution (8 mL) was then placed in a 10-mL syringe and maintained at 50 °C by the heating block of the rapid prototyping (RP) device (EZ-ROBO5, Iwashita, Japan). The PCL solution was connected with an adapter assembly syringe in an automatic dispenser (AD 3000C, Iwashita Engineering, Inc., Tokyo, Japan), operated by an air compressor to check the extrusion of the solution. The speed and air pressure were set at 5 mm sec^−1^ and 55 kPa, respectively. The syringe (25 gauge) was immersed in a dish containing 200 mL of ethanol. Then, layer-by-layer porous PCL scaffolds were fabricated by operating the RP device. The fabricated PCL scaffolds immersed in ethanol were hardened and organic solvents within the scaffold, including acetone and DMSO, were removed in the ethanol solution. Then, the PCL scaffolds were air-dried and further lyophilized for three days to remove the residual organic solvents. The fabricated PCL scaffolds were cylindrical (dimensions: 4 mm height × 8 mm diameter).

### 4.3. Fabrication of BMP-2/TA/PCL Scaffolds

In order to modify a PCL scaffold using TA and BMP-2, a PCL scaffold was placed in a PBS solution (pH 7.4) containing TA (10 µg) and allowed to react under gentle stirring. After 12 h reaction, the supernatant PBS solution was harvested to determine the TA content on the PCL scaffold, and the PCL scaffold was washed three times with fresh PBS solution and lyophilized for three days to yield a TA-coated PCL (TA/PCL) scaffold. The TA coating on the scaffold surface was quantified by the bicinchoninic acid (BCA) assay according to the manufacturer’s protocols. The collected supernatant PBS was incubated in the BCA working reagent for 1 h at 37 °C and its absorbance was recorded at 562 nm using a Flash Multimode Reader (Varioskan™, Thermo Scientific, Waltham, MA, USA). The TA content on the scaffold surface was determined based on the absorbance difference between original TA solution and the collected supernatant PBS after coating and a calibration curve drawn with standard TA solutions. The coating efficiency (%) of TA on the scaffold surface was calculated by the coated amount of TA over the initial TA amount.

To further immobilize the BMP-2 on PCL or TA/PCL, the scaffolds were immersed in a PBS solution (pH 7.4), followed by the addition of BMP-2 (500 ng mL^−1^) under gentle stirring overnight. After collecting the supernatant PBS solution, BMP-2-immobilized PCL or TA/PCL scaffolds were then washed several times with distilled water (DW) and lyophilized for three days to obtain BMP-2-immobilized PCL (BMP-2/PCL) or BMP-2-immobilized TA/PCL (BMP-2/TA/PCL). To quantify the immobilized amount of BMP-2 on the scaffold surface, BMP-2 in the supernatant was measured with an ELISA kit. The amount of immobilized BMP-2 on the scaffold surface was calculated as a concentration difference between the original BMP-2 solution and the supernatant solution. The immobilization efficiency (%) of BMP-2 on the scaffold surface was calculated by the immobilized amount of BMP-2 over the initial BMP-2 amount.

### 4.4. Characterization of PCL and Modified PCL Scaffolds

The morphologies of the scaffolds, including PCL, TA/PCL, BMP-2/PCL, and BMP-2/TA/PCL, were observed by using scanning electron microscopy (SEM, S-2300, Hitachi, Tokyo, Japan). Each sample was gold-coated using a sputter-coater (Eiko IB, Tokyo, Japan). The surface compositions of these scaffolds were investigated by X-ray photoelectron spectroscopy (XPS) on a K-alpha spectrometer (ESCALAB250 XPS System, Theta Probe AR-XPS System, Thermo Fisher Scientific, Loughborough, UK) with an Al Kα X-ray source (1486.6 eV photons) at the Korean Basic Science Institute Busan Center.

### 4.5. SEM Observation of Cell Infiltration into the Scaffolds

To observe the cell infiltration into the scaffolds, the mouse osteoblastic MC3T3-E1 cells (1 × 10^6^ cells/scaffold, American Type Culture Collection, Manassas, VA, USA) were seeded on the scaffold. After 24 h incubation, the scaffold was harvested and fixed with 2.5% glutaraldehyde (Junsei Chemical Ltd., Tokyo, Japan) at 4 °C for one day. Then, the scaffold was rinsed with PBS and dehydrated in an ethanol/DW mixture from 50 to 100% in steps of 10% for 100 min per each step, followed by freeze-drying. To observe the cell morphology and attachment inside the scaffold, the freeze-dried scaffold was immersed into liquid nitrogen and cut in half with a knife. After coating with Pt, the attachment and morphologies of the cells inside the scaffold were observed by SEM (SEM, S-2300, Hitachi, Japan).

### 4.6. In Vitro BMP-2 Release Study

To determine the amount of BMP-2 released from BMP-2/PCL or BMP-2/TA/PCL, each sample was placed in a 50-mL tube (Falcon, Corning, NY, USA) containing PBS (pH 7.4) at 100 rpm at 37 °C. At pre-designed time intervals, PBS was collected and replenished with fresh PBS solution. The released amount of BMP-2 from the scaffolds was determined by an ELISA kit according to the manufacturer’s instructions using a Flash Multimode Reader (Varioskan™, Thermo Scientific, Waltham, MA, USA) at a wavelength of 450 nm.

### 4.7. Anti-Oxidant Studies

#### 4.7.1. Anti-Oxidant Activity Assay

To measure the total anti-oxidant activities of PCL, TA/PCL, BMP-2/PCL, and BMP-2/TA/PCL, a 2,2-diphenyl-1-picrylhydrazyl (DPPH) assay was performed as described in the previous study [48]. Each sample was immersed in PBS (pH 7.4) at 37 °C. After 24 h, 1 mL of the extract from each sample was applied with 3 mL of a 0.3 mM DPPH solution in methanol. The resulting content was mixed vigorously. The reaction mixture was kept in the dark for 30 min. The absorbance of the reaction mixture was measured at 517 nm using a Flash Multimode Reader. The lower absorbance represents higher DPPH scavenging activity. The scavenging activity of the sample was calculated as follows: Percentage of DPPH scavenging = ((A_B_−A_S_)/A_B_) × 100. Here, A_B_ is the absorbance value of the blank solution (0.3 mM DPPH solution) and A_S_ is the absorbance value of the sample.

#### 4.7.2. Measurement of ROS in the Cell Level

The ROS scavenging abilities of each sample was evaluated by 2’,7-dichlorodihydrofluorescein diacetate (DCFDA) staining or DCFDA assay. In brief, MC3T3-E1 cells (mouse calvaria-derived preosteoblast, 1 × 10^5^/well) were seeded on the cover glass in 24-well plates and allowed to adhere. After 24 h, the cells were treated with 300 µM H_2_O_2_ at 37 °C for 30 min. Then, the harvested extracts from each scaffold in the DMEM medium without FBS (pH 7.4) for 24 h were treated to the cells for 6 h or 24 h. The cells were then stained with DCFDA (25 µM) for 45 min under dark conditions, washed with PBS, and fixed with 2.5% paraformaldehyde for 30 min. Fluorescence images of each group were obtained using a confocal laser scanning microscope (LSM 700, Carl Zeiss, Jena, Germany). To further quantify the ROS levels in cells treated with the extracts from each scaffold, a DCFDA (Ex/Em = 485 nm/535 nm) cellular ROS detection assay kit (Abcam, Cambridge, MA, USA) was used according to the manufacturer’s protocols. The quantitative fluorescence intensities were measured using a Flash Multimode Reader.

#### 4.7.3. Protection of Cell Viabilities against the ROS Environment

Cell viabilities of MC3T3-E1 cultured on each scaffold under ROS environments were measured using the CCK-8 kit. In brief, the cells (1 × 10^5^ cells/well) were seeded on each scaffold or tissue culture polystyrene (TCPS) in a 24-well plate and allowed to adhere. After 24 h, each scaffold with cells was incubated using a culture medium containing 300 µM H_2_O_2_ at 37 °C for 30 min, followed by further incubation for 6 h or 24 h. Then, the cells on each scaffold were treated with CCK-8 reagent solution for an additional 1 h. The reagent was carefully transferred to 96-well plates and the optical density of each group was measured at 450 nm using a Flash Multimode Reader.

### 4.8. Anti-Inflammatory Effects

In order to evaluate the in vitro anti-inflammatory effects of the scaffolds on LPS-induced MC3T3-E1 cells, the mRNA levels of pro-inflammatory factors, including MMP-3, COX-2, IL-6, and TNF-α, were measured by the real-time polymerase chain reaction (real-time PCR). MC3T3-E1 cells (1 × 10^5^ cells/mL/well) were seeded on each scaffold and incubated. After 24 h, LPS (1 µg/mL) was treated to the cells in each group. At one day and three days, the cells in each group were rinsed and harvested for total RNA isolation. Total RNA was extracted using an RNeasy Plus Mini Kit (Qiagen, Valencia, CA, USA), according to the manufacturer’s instructions. The RNA concentration was measured using a NanoDrop spectrometer (ND-1000 spectrometer, NanoDrop Technologies, Inc., Wilmington, DE, USA). Total RNA (1 μg) was reverse-transcribed into cDNA using AccuPower RT PreMix (Bioneer, Daejeon, Korea) according to the manufacturer’s protocols. All PCR amplifications were performed using AccuPower PCR PreMix (Bioneer, Daejeon, Korea). The primer sequences of the targeted genes were as follows: MMP-3, (F) 5′-ACC TGT CCC TCC AGA ACC TG-3′, (R) 5′-AAC TTC ATA TGC GGC ATC CA-3′; COX-2, (F) 5′-CAG CCA TAC TAT GCC TCG GA-3′, (R) 5′-GGA TGT CTT GCT CGT CGT TC-3′; IL-6, (F) 5′-CCG TTT CTA CCT GGA GTT TG-3′, (R) 5′-GTT TGC CGA GTA GAC CTC AT-3′; and TNF-α, (F) 5′-CTC CCA GAA AAG CAA GCA AC-3′, (R) 5′-CGA GCA GGA ATG AGA AGA GG-3′. PCR amplification and detection were performed using an ABI7300 Real-Time Thermal Cycler (Applied Biosystems, Foster City, CA, USA). The levels of the targeted genes, including MMP-3, COX-2, IL-6, and TNF-α, were normalized to those of glyceraldehyde 3-phosphate dehydrogenase (GAPDH) and expressed as relative values.

### 4.9. ALP Activity and Calcium Deposition

For the evaluation of the in vitro osteogenic effects of each scaffold, MC3T3-E1 cells (1 × 10^5^ cells/mL/well) were seeded on each scaffold in 24-well tissue culture plates and maintained in DMEM, supplemented with 10% FBS and 1% antibiotic. At three days and nine days, the cells were rinsed and lysed with 1× RIPA buffer. The lysates were centrifuged at 13,500 rpm for 1 min at 4 °C. The supernatants were then mixed with *p*-nitrophenyl phosphate solution, and the resulting solution was maintained for 30 min at 37 °C. The reaction was stopped by adding 500 μL of 1 *N* NaOH. ALP activity was determined by measuring conversion of *p*-nitrophenyl phosphate to *p*-nitrophenol by optical density at 405 nm, determined using a Flash Multimode Reader.

To investigate calcium deposition of the cells treated with each scaffold, MC3T3-E1 cells (1 × 10^5^ cells/mL/well) were carefully seeded on each scaffold. At seven days and 21 days, the cells were washed with PBS and 0.5 *N* HCl (500 μL) was added to the cells. After centrifugation at 13,500 rpm for 1 min, the supernatant was used for calcium deposition measurements using the QuantiChrom™ Calcium Assay Kit (DICA-500, BioAssay Systems, Hayward, CA, USA) according to the manufacturer’s instructions. Based on the standard of calcium chloride, the amount of calcium deposited was determined by a microplate reader at 612 nm.

### 4.10. Statistical Analysis

Quantitative data are presented as the mean ± the standard deviation, and statistical comparisons were performed via one-way ANOVA using SYSTAT software (Chicago, IL, USA). Differences were considered to be statistically significant at * *p* < 0.05 and ** *p* < 0.01.

## 5. Conclusions

In this study, we fabricated a new 3D PCL scaffold with TA and BMP-2 (BMP-2/TA/PCL) using the inherent multiple interactions of TA with various substrates and proteins. BMP-2/TA/PCL is able to effectively scavenge ROS in cells, protect MC3T3-E1 cells from the toxic ROS environment, and significantly suppress the mRNA levels of pro-inflammatory cytokines in inflamed cells. Furthermore, due to the sustained and controlled release of BMP-2 from the scaffolds, the BMP-2/TA/PCL scaffold stimulated osteogenic differentiation of MC3T3-E1 cells by increasing ALP activity and calcium deposition. We suggest that this TA coating method on the scaffold is useful for biofunctionalization of the scaffolds without loss of drug activity. Additionally, the fabricated BMP-2/TA/PCL scaffold will have potential for bone tissue regeneration.

## Figures and Tables

**Table 1:** Surface elemental compositions of PCL and modified PCL scaffolds determined by X-ray photoelectron spectroscopy (XPS).

Samples	C1s (%)	N1s (%)	O1s (%)	Total (%)
PCL	77.64	-	22.36	100
TA/PCL	76.41	-	23.59	100
BMP-2/PCL	76.68	0.64	22.68	100
BMP-2/TA/PCL	74.49	1.28	24.23	100
